# International Spread of Multidrug-resistant *Salmonella* Schwarzengrund in Food Products

**DOI:** 10.3201/eid1305.061489

**Published:** 2007-05

**Authors:** Frank M. Aarestrup, Rene S. Hendriksen, Jana Lockett, Katie Gay, Kathryn Teates, Patrick F. McDermott, David G. White, Henrik Hasman, Gitte Sørensen, Aroon Bangtrakulnonth, Srirat Pornreongwong, Chaiwat Pulsrikarn, Frederick J. Angulo, Peter Gerner-Smidt

**Affiliations:** * Danish Institute for Food and Veterinary Research, Copenhagen, Denmark;; † Centers for Disease Control and Prevention, Atlanta, Georgia, USA; ‡ Atlanta Research and Education Foundation, Atlanta, Georgia, USA; § US Food and Drug Administration, Laurel, Maryland, USA; ¶ Ministry of Public Health, Bangkok , Thailand; # Statens Serum Institut, Copenhagen, Denmark

**Keywords:** Salmonella enterica serovar Schwarzengrund, antimicrobial drug resistance, Thailand, Denmark, United States, pulsed-field gel electrophoresis, research

## Abstract

We compared 581 *Salmonella enterica* serotype Schwarzengrund isolates from persons, food, and food animals in Denmark, Thailand, and the United States by antimicrobial drug susceptibility and pulsed-field gel electrophoresis (PFGE) typing. Resistance, including resistance to nalidixic acid, was frequent among isolates from persons and chickens in Thailand, persons in the United States, and food imported from Thailand to Denmark and the United States. A total of 183 PFGE patterns were observed, and 136 (23.4%) isolates had the 3 most common patterns. Seven of 14 isolates from persons in Denmark had patterns found in persons and chicken meat in Thailand; 22 of 390 human isolates from the United States had patterns found in Denmark and Thailand. This study suggests spread of multidrug-resistant *S.* Schwarzengrund from chickens to persons in Thailand, and from imported Thai food products to persons in Denmark and the United States.

## Abstract 2

This serovar was isolated from persons, food, and food animals in Thailand, Denmark, and the United States.

*Salmonella enterica* is a common cause of human gastroenteritis and bacteremia worldwide, and a wide variety of animals, particularly food animals, have been identified as reservoirs for nontyphoidal *Salmonella* spp. (*1*–*3*). Human infections with nontyphoidal *Salmonella* are commonly caused by ingestion of food that has been contaminated by animal feces (*3*). Although >2,500 serovars of *S. enterica* have been identified, most human infections are caused by a limited number of serovars. *S.* serovar Typhimurium and serovar Enteritidis are the most common causes of human salmonellosis worldwide, although other serovars have been reported to be more prevalent in some regions (*3*–*7*). Shifts in prevalence of specific strain types and serovars can reflect the influence of international travel and trade of animals and food products, and can therefore serve as useful epidemiologic markers.

We recently reported an increase in the prevalence of *S.* serovar Schwarzengrund in broiler chickens in Thailand and an increase in the proportion of human *Salmonella* infections caused by *S.* Schwarzengrund in Thailand (*6*), from 0% in 1992 to 2.4% in 2001. This serovar was also recently reported as causing more illness in Denmark (www.germ.dk) and the United States, where several isolates have shown multidrug resistance (*8*,*9*).

In recent years, an increase in antimicrobial drug resistance, including resistance to nalidixic acid, among *Salmonella* spp. has been observed in many countries, particularly in Asia (*10*–*17*). Nalidixic acid–resistant and ciprofloxacin-resistant *S.* Schwarzengrund has been reported in Taiwan (*18*) and the United States (*9*), the US cases linked to patients previously hospitalized in the Philippines. The emergence of antimicrobial drug resistance is a matter of concern. Persons with infections caused by antimicrobial drug–resistant *Salmonella* infections, particularly nalidixic acid–resistant *Salmonella* infection, are more likely to die, are more likely to be hospitalized, and are hospitalized for longer periods than patients with infections caused by susceptible strains (*18*–*20*).

Antimicrobial drug susceptibility profiles and genetic strain typing methods are useful epidemiologic tools to determine the sources of infections, including potential links between food animals and persons. Pulsed-field gel electrophoresis (PFGE) is highly discriminatory and useful in epidemiologic studies (*21*,*22*). To our knowledge, no molecular studies on *S.* Schwarzengrund have been previously described.

This study was conducted to determine the clonality and molecular variation of *S.* Schwarzengrund from persons, food products, and animals in Denmark, Thailand, and the United States. In addition, antimicrobial drug resistance profiles were determined for some of the isolates. The implications of the findings in relation to the global spread of new serovars and the potential international spread by imported food products are discussed.

## Materials and Methods

### Bacterial Isolates

A total of 581 *S.* Schwarzengrund isolates were included, 73 from Denmark, 105 from Thailand, and 403 from the United States. All available isolates were selected from the strain collections at the Danish Institute for Food and Veterinary Research (n = 59) and Statens Serum Institut in Denmark (n = 14); the World Health Organization International *Salmonella* and *Shigella* Centre in Thailand (n = 105); and the US Food and Drug Administration (n = 7) and Centers for Disease Control and Prevention (CDC) in the United States (n = 396) (Table).

**Table:** Origin and occurrence of resistance among *Salmonella enterica* serovar Schwarzengrund isolates from humans, food, and food animals in Denmark, Thailand, and the United States

Country/source		No. isolates tested/ total no. isolates	No. isolates resistant to antimicrobial drugs*							
			AMP	CHL	CIP	GEN	NAL	STR	SUL	TET
Denmark										
	Humans	14/14	8	3	1	6	8	11	9	8
	Pigs on farm	22/22	0	2	0	0	0	4	2	2
	Pork	4/4	0	0	0	0	0	0	0	0
	Chicken meat of unknown origin	7/7	4	1	0	4	5	5	6	4
	Imported chicken	13/13	11	0	0	11	11	12	13	13
	Imported turkey	9/9	1	0	0	1	1	4	4	4
	Others	0/4	–	–	–	–	–	–	–	–
Thailand										
	Humans†	46/57	30	13	10	27	42	45	41	35
	Chicken meat	44/48	23	16	2	28	39	39	36	23
	Turkey meat	2/4	0	0	0	0	0	0	0	0
United States										
	Humans	38/390	13	08	16	4	17	4	20	22
	Chicken meat	0/3	–	–	–	–	–	–	–	–
	Turkey on farm	0/1	–	–	–	–	–	–	–	–
	Pigs on farm	2/2	0	0	0	0	0	0	1	1
	Imported food	3/3	1	2	1	2	3	2	3	3
	Total	204/581	91	45	30	83	126	126	135	115

*AMP, ampicillin; CHL, chloramphenicol; CIP, ciprofloxacin; GEN, gentamicin; NAL, nalidixic acid; STR, streptomycin; SUL, sulfamethoxazole; TET, tetracycline. †One human isolate from Thailand was resistant to ceftiofur.

The 73 isolates from Denmark were isolated from 1995 to 2004: 14 from ill persons, ≥2 of whom reported travel to Thailand in the 30 days before specimen collection; 22 from pigs on farms; 20 from chicken meat; 9 from turkey meat; 4 from pork; and 4 from other food sources. Of the 20 chicken meat products tested in Denmark, ≥13 were imported, and 10 of these were known to be from Thailand. The origin of the remaining 7 chicken meat products was not known. The 105 isolates from Thailand were isolated from 1994 to 2003 and included 57 from ill persons at 17 different medical facilities and 48 from chicken meat. The 403 isolates from the United States were isolated from 1998 to 2005: 390 from ill persons, 4 from turkey meat, 3 from chicken meat, 2 from pigs on farms, 1 from a turkey on a farm, 1 from a squid roll imported from Taiwan, 1 from a catfish imported from Thailand, and 1 from a dehydrated whole chili imported from Thailand. Most isolates from Denmark and Thailand, but only a limited number of isolates from the United States, were available for susceptibility testing.

### Antimicrobial Drug Susceptibility

Of the 581 isolates obtained, 204 were tested for antimicrobial drug susceptibility: 69 from Denmark, 90 from Thailand, and 45 from the United States. Susceptibility to antimicrobial agents was performed as MIC determinations by using a commercially prepared, dehydrated panel (Sensititer; TREK Diagnostic Systems Ltd., East Grinstead, UK), according to the Clinical and Laboratory Standards Institute/National Committee for Clinical Laboratory Standards (*23*) for the following antimicrobial agents: ampicillin, ceftiofur, chloramphenicol, ciprofloxacin, gentamicin, nalidixic acid, streptomycin, sulfamethoxazole, and tetracycline. Reduced susceptibility to ciprofloxacin was defined as MIC ≥0.125 mg/L and resistance as MIC ≥4 mg/L.

### Pulsed-Field Gel Electrophoresis

All 581 isolates were analyzed for genetic relatedness by PFGE by using *Xba*I according to the CDC PulseNet protocol (*24*). Electrophoresis was performed with a CHEF-DR III System (Bio-Rad Laboratories, Hercules, CA, USA) by using 1% SeaKem agarose in 0.5× Tris-borate-EDTA at 180 V. Running conditions consisted of 1 phase from 2.2 to 63.8 s for a run time of 22 h.

All isolates from Denmark and Thailand and 7 of the food isolates from the United States were typed at the Danish Institute for Food and Veterinary Research; the remaining isolates from the United States were typed by PulseNet-participating state and local health departments, with the PFGE patterns submitted to the PulseNet database. One representative of each PFGE type identified in Denmark was sent to CDC for comparison with the PulseNet database. Comparison of the PFGE profiles was performed by using Bionumerics software v3.5 (Applied Maths, Sint-Martens-Latem, Belgium).

## Results

### Antimicrobial Drug Susceptibility

Among the 69 isolates from Denmark, nalidixic acid resistance was found in 8 (57%) of 14 human isolates, including those from both patients with known recent travel to Thailand, and in 16 (80%) of 20 chicken isolates, including all 10 isolates from chicken imported from Thailand. Nalidixic acid resistance was not found in pig, pork, or turkey meat isolates (Table). All nalidixic acid–resistant isolates from Denmark also displayed reduced susceptibility to ciprofloxacin. Nalidixic acid resistance was common among isolates from Thailand, including 42 (91%) of 46 human isolates and 39 (89%) of 44 chicken meat isolates. All nalidixic acid–resistant isolates from Thailand also exhibited reduced susceptibility to ciprofloxacin; 12 isolates (10 from persons and 2 from chickens) were resistant to ciprofloxacin. Ten of the ciprofloxacin-resistant isolates from Thailand contained 2 single-base substitutions in the *gyrA* gene at codons83 [(T**C**C (Ser) → T**T**C (Phe)) and 87 (**G**AC (Asp) → **A**AC (Asn)]. (Mutated bases are shown in boldface.)

Among the 45 isolates from the United States for which susceptibility results were available, nalidixic acid resistance was found in 17 (45%) of 38 human isolates and 3 (43%) of 7 food and food animal isolates, including all 3 isolates from imported food from Thailand and Taiwan. All nalidixic acid–resistant isolates from the United States also showed decreased susceptibility to ciprofloxacin; 16 of the 17 nalidixic acid–resistant isolates from persons and the isolate from an imported dehydrated whole chili from Thailand were resistant to ciprofloxacin.

### Pulsed-Field Gel Electrophoresis

A total of 180 unique PFGE patterns were observed among the 581 isolates, with 136 (23%) isolates having the 3 most common patterns (Appendix Figure). The most common pattern (58 isolates, JM6X01.0001) represented only human isolates from the United States; 5 of these isolates were susceptibility tested, and all were resistant to sulfamethoxazole and tetracycline and susceptible to all other antimicrobial agents tested. The second most common pattern (44 isolates, JM6X01.0091) included isolates from persons in Denmark, chicken meat imported from Thailand to Denmark, and persons and chickens in Thailand; all 44 isolates were susceptibility tested, and 42 (95%) were nalidixic acid resistant. The third most common pattern (34 isolates, JM6X01.0015) included isolates from persons and chicken meat in Denmark, persons and chicken meat in Thailand, persons in the United States, and catfish imported from Thailand to the United States; 20 of these isolates were tested for susceptibility, and 15 (75%) were nalidixic acid resistant.

Two additional patterns are noteworthy. One pattern (18 isolates, JM6X01.0059) included 1 isolate from a person in Denmark, 5 isolates from chicken and 9 isolates from persons in Thailand, 2 isolates from persons in the United States, and the isolate from the dehydrated whole chili imported from Thailand to the United States. Of the 9 isolates that underwent susceptibility testing, 7 (78%) were ciprofloxacin-resistant. Another pattern (14 isolates, JM6X01.004) included only human isolates from the United States; 10 of these isolates were susceptibility tested, and all were ciprofloxacin-resistant. This is the pattern from the previously described outbreak of ciprofloxacin-resistant *S.* Schwarzengrund infections in medical facilities in Oregon (*9*).

Twenty-six different PFGE types were found among the 73 isolates from Denmark (Appendix Figure). Of these, 11 were found among 14 human isolates, 10 among the 26 isolates from pigs and pork, 7 among the 19 chicken meat isolates, and 4 among the 9 turkey meat isolates. Seven (50%) human isolates belonged to types that were also found in food, including 6 PFGE types found in chickens. Forty-three different PFGE types were found among the 105 isolates from Thailand; 25 of these were found among the 57 human isolates and 25 among the 48 chicken meat isolates. Thirty-five (61%) of the human isolates and 30 (63%) of the chicken isolates belonged to PFGE types that were found in both sources.

Four of the 6 PFGE types, involving 7 of the 14 isolates found among persons and chickens in Denmark, were also observed among persons and chicken meat in Thailand. Both ill persons in Denmark who reported travel to Thailand in the 30 days before specimen collection were infected with 1 of the PFGE types common in Denmark and Thailand. These 4 PFGE types included the most common type among persons and chickens in Denmark and the 2 most common types among persons and chickens in Thailand.

Among the 403 isolates from the United States, 121 different PFGE types were found; 116 of these were found among the 390 human isolates and 12 among the 13 food and food animal isolates. Seven PFGE types were found in both persons and food; 73 (19%) of the human isolates and 8 (62%) of the food isolates belonged to types found in persons and food. All 8 food isolates with types also found in persons were found in foods imported to the United States, including food imported from Thailand. Twenty-two (6%) of the 390 human isolates from the United States matched PFGE patterns found in persons and chickens in Denmark or Thailand. Of the 19 isolates from persons in Denmark and the United States that belonged to PFGE types found in both countries and Thailand, 4 were tested for resistance and 3 (75%) of these were multidrug resistant.

## Discussion

Foodborne diseases caused by nontyphoidal *Salmonella* spp. represent an important public health problem worldwide. In Denmark alone, the costs related to foodborne cases of salmonellosis were estimated to be US $10.4 million to $25.5 million in 2001 (*25*). In the United States nontyphoidal *Salmonella* spp. are responsible for an estimated >1.4 million illnesses, almost 16,000 hospitalizations, and >500 deaths every year (*26*) at an estimated annual cost of up to $2.3 billion. (*27*).

Historically, *Salmonella* serotypes Enteritidis and Typhimurium have been the most important causes of nontyphoidal salmonellosis. *S.* Schwarzengrund is a less common cause of human salmonellosis worldwide. In recent years, however, the relative incidence of this serovar seems to have increased (*6*,*8*). It now ranks among the 20 most frequently identified *Salmonella* serovars in several countries, including Slovakia, New Zealand, Venezuela, and Thailand; is among the 40 most frequently identified serovars in Denmark and the United States; and was the fifth most common serovar isolated from retail meat in the United States in 2004, associated exclusively with poultry products. Other studies also suggest that poultry could be the most common reservoir (*6*,*28*,*29*).

This study showed a high frequency of antimicrobial drug resistance, including an unusually high prevalence of nalidixic acid resistance, among *S.* Schwarzengrund isolates from chickens in Denmark, persons and chickens in Thailand, and food products imported into the United States. In contrast, the frequency of resistant isolates from pigs and turkey meat in Denmark was low. The prevalence of resistance among isolates from persons in Denmark was intermediate compared with the high level in persons and chickens in Thailand and the low level in Danish food animals. Along with the PFGE data, these resistance data support a hypothetical transmission of *S.* Schwarzengrund from chickens to persons in Thailand, and transmission from chickens, pigs, and turkeys to persons in Denmark.

Ciprofloxacin resistance was detected in 29 (24%) of 123 nalidixic acid–resistant *S.* Schwarzengrund isolates. Fluoroquinolone (ciprofloxacin) resistance among *Salmonella* spp. has recently emerged in several countries (*30*–*32*). Ten ciprofloxacin-resistant isolates tested in this study contained double mutations in *gyrA* at codons 83 (Ser → Phe) and 87 (Asp → Asn). These positions, located in the quinolone resistance–determining region of *gyrA*, are commonly reported among numerous bacterial species, including *Salmonella* isolates with a high-level of ciprofloxacin resistance (*30*–*32*). A ciprofloxacin-resistant strain of *S.* Schwarzengrund recently caused a nosocomial outbreak involving 2 nursing homes and 1 hospital in Oregon (*9*). The index patient in the Oregon outbreak was initially hospitalized in the Philippines. In many countries, including Denmark, fluoroquinolones are the drugs of choice for treating complicated gastrointestinal infections. Thus, resistance to this group of antimicrobial agents is especially critical, both for management of salmonellosis and because of the association of resistance with increased illness and death (*19*,*20*).

To our knowledge, this is the first study of the molecular epidemiology of *S.* Schwarzengrund. The study demonstrated a substantial diversity in PFGE patterns of this serotype with the presence of several common international clones. The PFGE types of isolates from persons and chicken meat in Thailand formed overlapping populations, with more than half of the isolates from both sources belonging to shared types. This supports the involvement of chicken meat as a reservoir for human *S.* Schwarzengrund infections in Thailand.

The epidemiology of *S.* Schwarzengrund infections in Denmark is complicated. Although some PFGE types were only found among isolates from pigs, several PFGE types were shared among isolates from persons and pigs; persons, chicken meat, and turkey meat; and persons and chicken meat. This suggests that chicken meat, pork, and turkey meat are sources of *S.* Schwarzengrund infections for persons in Denmark. Because modern trade and distribution of food products makes it difficult to determine the country of origin of meat samples sold retail in Denmark, the sources of the chicken meat, pork, and turkey meat included in this study are not completely known. However, Denmark has been importing an increasing amount of chicken meat, and much of this imported chicken is from Thailand. In addition, *S.* Schwarzengrund has, to our knowledge, not been detected in the Danish production of chicken. In this study, ≥13 of the 20 chicken meat products tested in Denmark were imported, and 10 of these were from Thailand. These data, with the identification of identical PFGE types from persons and chickens in both Denmark and Thailand, support the possibility that some persons in Denmark acquired *S.* Schwarzengrund from imported chicken meat from Thailand. Another less frequent means of acquiring the infections is travel by persons from Denmark to Thailand; ≥2 of the 14 case-patients in Denmark had recently returned from travel to Thailand before they became ill.

A limited number of isolates from food and food animals in the United States were included in this study. The prevalence of *S*. Schwarzengrund in retail meat was low in 2003, with only 3 isolates recovered. In addition, PFGE data on isolates from food animals were not available for comparison in this study. The study would have benefited from additional isolates, especially from food and food animals in the United States. Nevertheless, a high proportion of these available isolates from food and food animals shared PFGE patterns with human isolates. In addition, several patterns found among human isolates in the United States were also present among human isolates in Denmark and Thailand, which suggests an international spread of these clones. Specifically, 1 PFGE type that was frequently ciprofloxacin resistant was found in a person in Denmark, in persons and chicken meat in Thailand, and in persons and the dehydrated whole chili imported from Thailand to the United States. Another PFGE type that was nalidixic acid–resistant was found in persons and chicken meat in Denmark, persons in the United States, and in catfish imported from Thailand to the United States.

In our study and other studies, *Salmonella* isolates from imported food in Denmark had a higher frequency of resistance than was found in domestically produced meats (*33*). A study from the United States also reported a high frequency of antimicrobial drug resistance among *Salmonella* isolates from imported food (*34*). Food is an important vehicle for the national and international dissemination of *Salmonella* spp. and antimicrobial drug resistance genes from food animals to persons (*35*–*38*).

This study supports the conclusion that multidrug-resistant, including nalidixic acid–resistant, *S.* Schwarzengrund was likely disseminated internationally by chicken products from Thailand. Because antimicrobial drug resistance among *Salmonella* isolates from food animals commonly reflects antimicrobial drug use in food animals, efforts are needed to ensure appropriate use of antimicrobial agents in food animals and to improve food safety to reduce dissemination of *Salmonella* spp. worldwide.

## Supplementary Material

Appendix Figure Pulsed-field gel electrophoresis types among Salmonella enterica serovar Schwarzengrund isolates from various sources in Denmark, Thailand, and the United States.
