# Supplementary material for: International Spread of Multidrug-resistant Salmonella Schwarzengrund in Food Products
Source: Emerg Infect Dis. 2007 May;13(5):726–31. doi: 10.3201/eid1305.061489 (PMC2738437; doi:10.3201/eid1305.061489)
Supplement: Appendix Figure — Pulsed-field gel electrophoresis types among Salmonella enterica serovar Schwarzengrund isolates from various sources in Denmark, Thailand, and the United States. [file 06-1489_appF-s1.pdf]

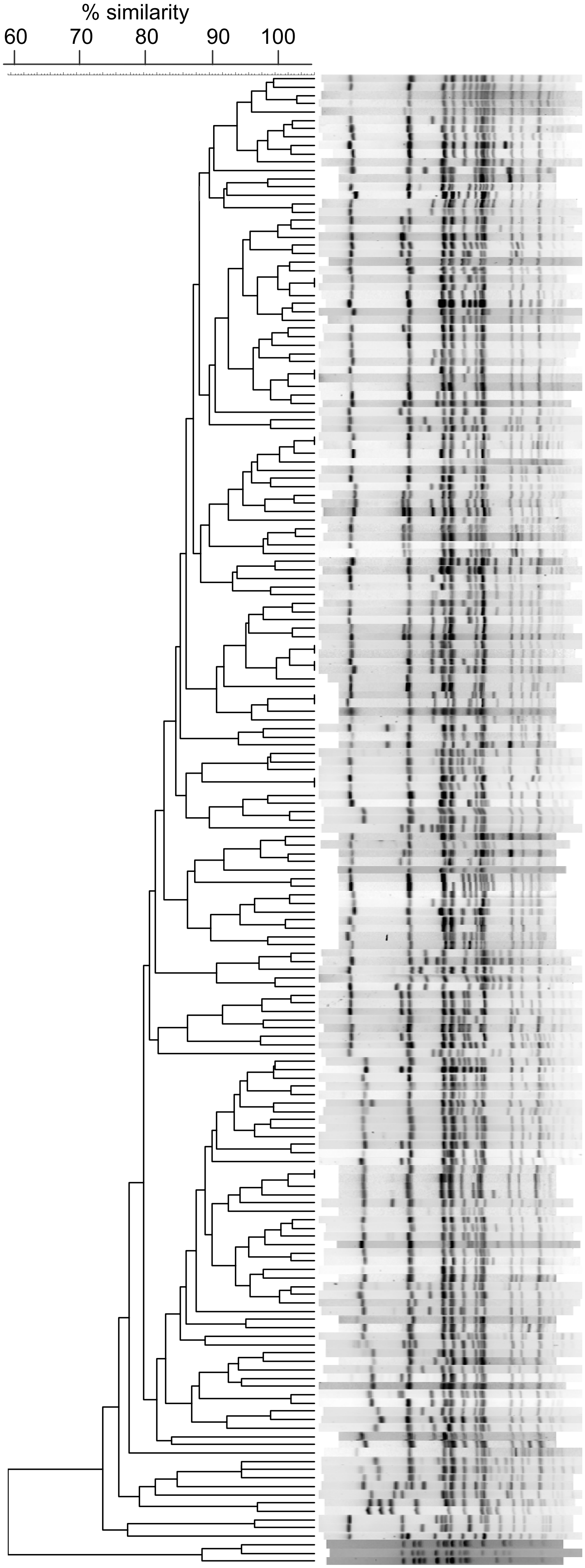

PulseNet-number JM6X01.-		Origin and number of isolates					
		USA	Denmark		Thailand		Row total
	Human	Food products	Human	Food products/ animals	Human	Food products	
0058					1		1
0011	23	1					24
0161		1					1
0160	1	1					2
0037	4						4
0049	5						5
0038	15						15
0158	1						1
016	1						1
0164	3						3
0169	3						3
0139	1						1
0138	1						1
0165	1						1
0154	1						1
0166	1						1
0110	1						1
0053			1	2			3
0143	1						1
0050	2		1				3
0055				1			1
0056				1			1
0044	2						2
0054		1			3		4
0014	3						3
0142	1						1
0018	1						1
0012	2						2
0159	1						1
0150	1						1
0040	2						2
0015	14	1	2	4	8	5	34 ←
0146	2						2
0016	1						1
0147	1						1
0126	2						2
0034	16	1					17
0118		1					1
0033	1						1
0148	1						1
0072						1	1
0062					1		1
0063				1	1		2
0108	1						1
0031	1						1
0020	7						7
0117	1						1
0032	1						1
0109	1						1
0046	8						8
0023	5						5
0029	1						1
0030	2						2
0013	1						1
0127	1						1
0035	4						4
0131	1						1
0130		1					1
0167	1						1
0141	1						1
0125	1						1
0039	4						4
0123		1					1
0052	2						2
0048	24						24
0122	1						1
0128	1						1
0129	1						1
0066	1			1			2
0120	1						1
0065			1	2	1	2	6
0051	2						2
0119	1						1
0022	5						5
0144	1						1
0151	2						2
0047	2						2
0152	1						1
0064						1	1
0021	1						1
0140	2						2
0107					1		1
0060					1		1
0059	2	1	1		9	5	18 ←
0067				2			2
0068				1			1
0057			1				1
0145	1						1
0083						1	1
0082						1	1
0077				3			3
0134	1						1
0135	1						1
0133	1						1
0136	1						1
DFVF 4b				7	2		9
0024	4						4
0001	58						58 ←
0155	1						1
0028	15	2					17
0153	1						1
0027	2						2
0137	1						1
0156	1						1
0157	1						1
0036	17						17
0168	2						2
0081					1		1
0170	5						5
0045	4						4
0069				11			11
0070				2			2
0071				1			1
0061					1		1
0019	1						1
0073				1			1
0074					1		1
0116					1		1
0096					1		1
0043	1						1
0112	2						2
0094					1		1
0174	4						4
0099						1	1
0098					1		1
0091			3	12	14	15	44 ←
0095						1	1
0097						1	1
0093			1				1
0025	1						1
0100				1			1
0003	1						1
0007	2						2
0004	14						14 ←
0005	2						2
0090					1	1	2
0006	2						2
0111	1						1
0101						1	1
0089	1				1		2
DFVF 14a*						1	1
0102					1		1
0041	3						3
0026	2				1	1	4
0009	1						1
0075					1		1
0076						1	1
0175	2						2
0092			1				1
0002	3						3
0010	1						1
0104					1		1
0103						1	1
0084						1	1
0173	1						1
0080					1		1
0171	4						4
0172	1						1
0113			1				1
0149	1						1
0078						1	1
0079						1	1
0088						1	1
0176	2						2
0008	1						1
0162	2	1					3
0085						1	1
0086						1	1
0087					1	1	2
0115						1	1
0114			1				1
0017	3						3
0042	3						3
0105	1						1
0106	1						1
0132	1						1
DFVF 41c*				1			1
DFVF 41a*				4			4
DFVF 41b*				1			1

*, types not given a Pulsenet number; ←, types mentioned in the text.
